# Questionnaire Development of a Good Nurse and Better Nursing From Korean Nurses' Perspective

**DOI:** 10.1097/JNR.0000000000000393

**Published:** 2020-06-27

**Authors:** Mihyun PARK, Eun-Jun PARK

**Affiliations:** 1 PhD, RN, Associate Professor, College of Nursing, The Catholic University of Korea, Seoul, ROK; 2 PhD, RN, Professor, Department of Nursing, Konkuk University, ROK.

**Keywords:** surveys and questionnaires, validation studies, nurses, nursing, nursing care

## Abstract

**Background:**

The concepts of “good nurse” and “better nursing” have changed over time and should be investigated from the perspective of nurses.

**Purpose:**

The aim of this study was to develop and assess the psychometric properties of two questionnaires used to assess “good nurse” and “better nursing.”

**Methods:**

The interview data of 30 registered nurses (RNs) from a previous study were reviewed to develop the questionnaire items, and content validity was examined. One hundred seventeen RNs participated in a pilot survey for pretesting the constructs, 469 RNs participated in a main survey to explore these constructs using exploratory factor analysis (EFA), and 468 RNs participated in model refining and validation using confirmatory factor analysis.

**Results:**

After a critical review of RN interview data and content validity evaluation, 73 of 124 statements on “good nurse” and 56 of 57 statements on “better nursing” were selected. In the pilot survey, the number of items was reduced to 45 for both questionnaires using an EFA. In the main survey, EFA was used to load 34 items on the five factors of the good nurse questionnaire and 26 items on the three factors of the better nursing questionnaire. In the confirmatory factor analysis, to obtain better fitting models, the good nurse questionnaire consisted of 17 items on the five factors of collaboration, professional competency, self-efficacy, a sense of achievement, and compassion, whereas the better nursing questionnaire consisted of 16 items on the three factors of person-centered nursing, proactive nursing, and expertise in caring. The construct reliability, convergent validity, and discriminant validity of the questionnaires were achieved.

**Conclusions/Implications for Practice:**

The concept of “good nurse” from the perspectives of the nurses in this study was similar with those of patients in previous studies, while including individual traits such as sense of achievement. Better nursing is conceptualized with the exemplary performance of nursing focusing on the nature of nursing and leading excellence and power in clinical practice. The study findings inform what nursing education and workforce development should focus on for nursing to continuously progress. Furthermore, it is recommended that the concepts of a good nurse and better nursing be compared across different countries using the questionnaires.

## Introduction

What makes a good nurse? Although the concepts of a good nurse vary in the nursing literature, “good nursing” is often referred to among the public and nursing professionals. According to the virtue ethics of Aristotle, a good nurse is one who possesses essential virtues to perform a nurse's function well. Nurse virtues have changed through history and differed depending on the identified function of a nurse as nurses' identity changes responding to changes in healthcare. Although a univocal definition of a good nurse across time and location seems to be impractical, it still requires a continuous inquiry among nursing scholars.

Since the beginning of modern nursing, nurses' identity has changed from assistants of physicians to providers of professional care to patients. This change has led to a paradigmatic shift in the concept of a good nurse (Begley, 2010). According to historical reviews (Begley, 2010; Fry, 2004), the notion of a good nurse has changed from an etiquette-oriented perspective to an ethics-oriented perspective and from a vocation to a professional. The virtue of a good nurse in the Nightingale period included assisting physicians with loyalty, obedience, and modesty. These are no longer generally considered virtues of current professional nurses, especially in many developed countries. Rather, accountability and autonomy may be the essential virtues of today's nursing professionals (Begley, 2010; Fry, 2004).

Nursing scholars who study the virtues and concept of a good nurse today often inquire the view of patients. This current trend in nursing studies is desirable because nurses identify their primary role as promoting the well-being of patients. Thus, nursing outcomes should reflect patients' experience with nurses. Patients commonly described a good nurse as having virtues such as being compassionate, kind, respectful, honest, and responsible in addition to having professional knowledge and skills to promptly address their care needs (Gallagher et al., 2009; Rchaidia et al., 2009; Van der Elst et al., 2012). Although these virtues are common in both Western and Eastern countries, Asian patients reflect Asian values and culture in their perspectives of a good nurse. In Taiwanese (Chou et al., 2007) and Korean (Cho et al., 2006; Han et al., 2006) studies, patients expressed that a good nurse should treat them as family or as a relative, which reflects Asian culture recognizing their family members as ideal and primary caregivers. The patients in a Japanese study (Izumi et al., 2006) emphasized having good interpersonal relationship skills as a virtue of a good nurse, introducing the traditional meaning of the *kanji* character “Person-*hito.*” Differences in the essential virtues of nurses across time and place may promote further study on a good nurse in various societies.

Furthermore, the perspectives of a good nurse must be learned from not only patients but also nurses. There are differences and similarities in the perspectives between patients and nurses (Aydin Er et al., 2017; Catlett & Lovan, 2011; Kim et al., 2019). Some virtues of a good nurse such as being compassionate, respectful, and responsible and having professional knowledge and skills were shared between patients and nurses. However, unlike patients, nurses identified collaboration or commitment to a relationship with colleagues or an organization as the virtues of a good nurse (Catlett & Lovan, 2011; Kim et al., 2019). Nurses do not limit their role in a patient–nurse relationship, although both patients and nurses often emphasize virtues required for the relationship. Accordingly, the perspectives of nurses on a good nurse need to be explored in addition to those of patients. The concept of a good nurse would reveal the critical virtues of nurses who are responding to rapidly changing healthcare environments, including patients' expectations. Thus, nursing education and evaluation should be consistent with the critical virtues of nurses to enhance the quality of the nursing workforce.

Korean scholars conducted a qualitative study to learn nurses' perspectives of a good nurse and better nursing for the first time in 2015 (Um et al., 2017). They investigated the concept of better nursing to develop a good nursing practice in terms of positive change and improvement in nursing. The participating nurses were asked to describe some anecdotes in narrative form based on their direct or indirect experiences of being a good nurse and promoting a better nursing practice. In the study, the definition of a good nurse or better nursing was not given to the nurses, and nurses were asked to provide their own perspectives by asking questions such as “Who is a good nurse?” and “What is your experience or observation about better nursing practice for patients?” The identified characters of a good nurse in the study were not very different from those in previous studies. For example, the nurses described good nurses as those who showed observation and assessment skills based on knowledge, compassion for their patients in pain and difficulties, and attitudes of helping or treating other nurses well. Better nursing was described using anecdotes of providing outstanding patient care with excellence and power, as emphasized in Benner (1984). For example, one of the study participants illustrated a caring situation that a nurse provided basic care to a patient in a coma in a comforting and skillful manner with a beautiful smile and soft words such as “Grandma, please enjoy your meal” every day although she had never received any response from this patient.

However, although this prior qualitative study informed about the characteristics of a good nurse and better nursing, it was limited in terms of the generalizability of findings. To obtain concepts of a good nurse and better nursing that are generally agreed upon by Korean nurses, this study surveyed a large number of nurses and explored these concepts using quantitative evidence. Furthermore, on the basis of the qualitative data of self-reflection in this study, two constructs of a good nurse and better nursing were developed and validated.

## Methods

### Study Design

This methodological study was designed to develop and validate two instruments for use in Korea. These instruments were (a) a good nurse questionnaire and (b) a better nursing questionnaire.

### Ethical Consideration

This study was approved by the institutional review board (No. MC16QISI0067). No individual identification information was collected, and the completed surveys were individually sealed and returned by mail.

### Study Procedure and Sample

**Figure 1:**
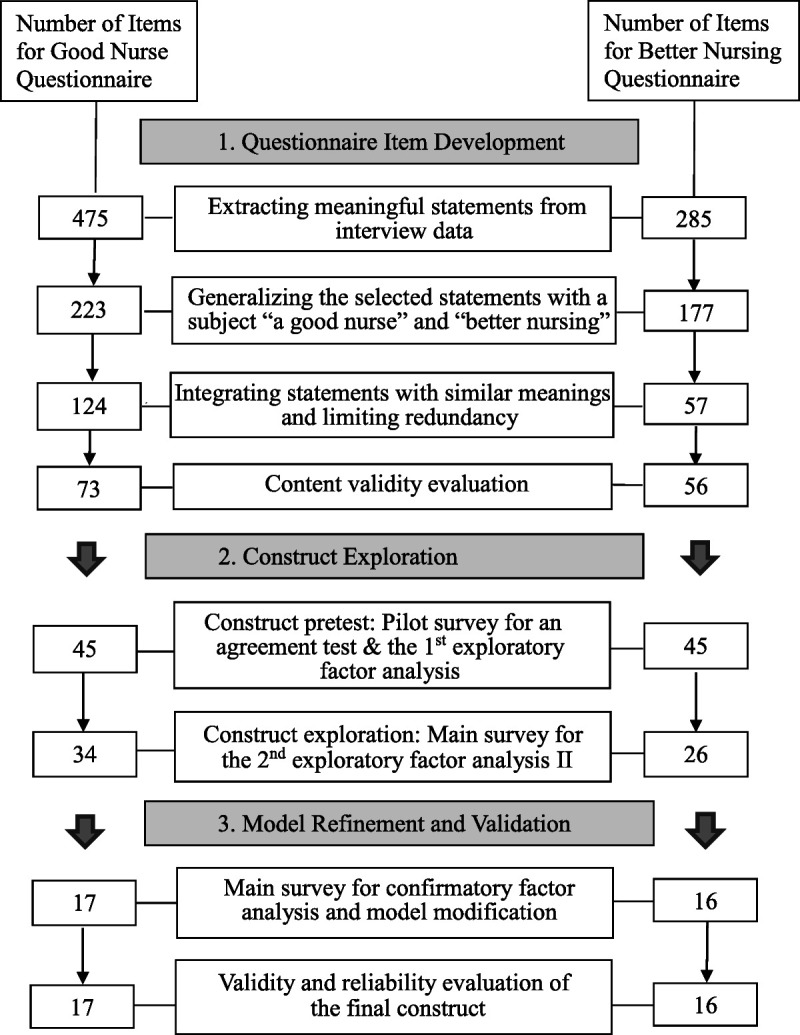
Study process

This study was conducted following the stages of a questionnaire-item development, repeated-construct exploration, and model refinement and validation (Figure 1). The English versions of the instruments were developed using forward–backward translation by bilingual experts.

The questionnaire items were developed based on the interview data of 30 registered nurses (RNs) and tested for content validity on 10 subject experts. The interview data were obtained from a previous study entitled, “A Good Nurse and Better Nursing,” in which one of the authors had participated (Um et al., 2017). Preliminary questionnaire items were developed using three steps. First, the interview data were imported to MAXQDA Version 11 (VERBI GmbH, Berlin, Germany), and all meaningful statements were selected, allowing for duplications, which resulted in 475 statements on a good nurse and 285 statements on better nursing. Second, the selected excerpts were restated into general statements applicable across different hospital settings and RNs. For example, a statement “When I did something wrong while taking care of my patient, I let the patients or colleagues know about it” was changed to “A good nurse frankly admits her/his errors and mistakes.” As a result, 223 statements on a good nurse and 177 on better nursing were obtained. Third, redundant statements with similar meanings were integrated and removed, whereas their meanings were clarified using the original data. For example, statements such as “A good nurse accepts even a patient's request that seems unnecessary” and “A good nurse admits and accepts a patient behavior that is annoying and difficult to understand” were combined into one statement of “A good nurse admits and accepts a patient's response that is difficult to understand because (s)he is a patient.” At the end of the questionnaire-item development process, there remained 124 statements on a good nurse and 57 on better nursing.

Ten subject experts evaluated the content validity of the questionnaire items using a 4-point scale. The experts were five nursing professors who had studied nursing humanities and five clinical nurses with clinical nursing careers of longer than 5 years each. Items with a content validity index of .80 or higher (Polit & Beck, 2006) were selected for the pilot survey. Four items with a content validity index of .70 were also included in the pilot survey, as they were repeatedly emphasized as critical characteristics of better nursing in the original interview data. Accordingly, the numbers of survey items for the pilot survey were 73 (58.9%) of 124 statements on a good nurse and 56 (98.2%) of 57 statements on better nursing.

The pilot survey was conducted to pretest the concept and shorten the survey. Perceptions of a good nurse and better nursing were evaluated on a scale ranging from 1 = *not agree at all* to 5 = *strongly agree*. An item was excluded from the first exploratory factor analysis (EFA) if it had less than an average score of 4.0 points. A sample size for factor analysis is at least 100 (Kyriazos, 2018). Thus, 117 of 125 invited RNs (response rate: 93.6%) participated in the pilot survey from four hospitals with 500 or more beds in August 2016. One hundred nine surveys were analyzed after excluding eight unreliable surveys because of most responses being uncompleted or answered without variation.

For the main survey, 1,040 RNs were invited and 950 (91.3% response rate) from 36 hospitals returned the individually sealed paper surveys by mail. These RNs were recruited in two stages. First, 40 hospitals were selected by cluster random sampling from all Korean hospitals grouped by four hospital locations and three hospital sizes (1,000 or more beds, 500–1,000 beds, and less than 500 beds). Second, 1,040 RNs were recruited by convenience sampling from the 40 hospitals.

Nine hundred thirty-seven surveys with reliable responses were selected and randomly assigned using MS Excel software for analysis in the construct exploration stage (*n* = 469) or the refinement and validation stage (*n* = 468). The participants' demographic characteristics, including age, gender, religion, duration of work, type of nursing unit, and work position, were not statistically different at α = .05 between the two sets of data.

### Data Analysis

SPSS Version 24.0 (IBM, Inc., Armonk, NY, USA) was used for the descriptive statistics analysis and EFA. AMOS 20.0 (IBM, Inc., Armonk, NY, USA) was used for the confirmatory factory analysis (CFA). In the repeated construct exploration stage, items with Pearson's correlation coefficients of either ≥ .80 or ≤ .30 were excluded from the factor analysis because of redundancy or low relevancy (Pett et al., 2003). Bartlett's test of sphericity and Kaiser–Meyer–Olkin (KMO) were calculated to evaluate the suitability of the data for EFA. Principal component analysis (PCA) was adopted for factor extraction, and orthogonal rotation (the varimax method) was applied assuming no correlation among factors. A factor loading of ≥ .50 was accepted. The number of factors was determined if the eigenvalue was greater than 1, percentage of extracted variance was ≥ 5%, and cumulative percentage of variance was ≥ 50% (Pett et al., 2003). The reliability of the questionnaire was assessed in terms of internal consistency using Cronbach's α coefficient. As pairwise deletion was adopted, sample sizes differed depending on the variables.

CFA was conducted using the AMOS 20.0 program to refine and validate the factor structure obtained as the result of EFA. Goodness-of-fit indices (GFIs) were adopted to test how well the construct structure from the EFA fits the validation data (*n* = 468). A chi-square test, normed χ^2^, root mean square error of approximation (RMSEA), standardized root mean squared residual (SRMR), GFI, and adjusted GFI (AGFI) were used as an absolute fit index, whereas normed fit index (NFI), comparative fit index (CFI), and Tucker–Lewis index (TLI) were used as an incremental index. The EFA factor structure was modified to improve model fit using the modification index (Kang, 2013).

The convergent validity of primitive constructs derived from the EFA results was accepted with standardized factor loading values of ≥ .50 and average variance extraction (AVE) values of ≥ .50 (Hair et al., 2010). Construct reliability, also called composite reliability, in CFA was considered acceptable at ≥ .70 (Hair et al., 2010). Discriminant validity was assessed using the criterion that the confidence interval of the estimated correlation between any two latent constructs (± 2 *SE*s from the point estimate) does not include 1 (Haddock & Maio, 2004).

## Results

### Construct Pretest Stage

In the pilot survey (*N* = 109), most of the participating RNs were female (95.4%, *n* = 104), were an average of 32.72 ± 6.48 years old, self-identified as religious (66.1%, *n* = 72), held a bachelor's degree (52.3%, *n* = 57), worked as a staff nurse (57.8%, *n* = 63), and had an average career duration of 10.26 ± 6.11 years. Before conducting a factor analysis, eight items of “a good nurse” and seven items of “better nursing” were removed because they had a low agreement level (< 4.0).

The KMO values were .90 for both instruments, indicating excellent sampling adequacy, and Bartlett's tests of sphericity were statistically significant (*p* < .001), rejecting the null hypothesis that no relationship exists among the items (Pett et al., 2003). Because all of the items were collapsed into one factor using PCA, a principal axis factoring was used for further item reduction. In the results of factor analysis with orthogonal rotation, items with communality < .50, with factor loadings < .30, or loaded on more than one factor were removed (Mooi, Sarstedt, & Mooi-Reci, 2018). After applying the criteria for factor retention such as the eigenvalue (> 1), percentage of extracted variance (≥ 5%), and cumulative percentage of variance (≥ 50%), 45 items under five factors were retained in the good nurse questionnaire and 45 items under three factors were retained in the better nursing questionnaire.

### Construct Exploration Stage

EFA was conducted with the first half of the main survey participants (*n* = 469 RNs). Most of the RNs in this group were female (95.7%, *n* = 449), were an average of 34.77 ± 9.26 years old, self-identified as religious (51.2%, *n* = 240), worked as a staff nurse (81.9%, *n* = 384), and had an average career duration of 11.33 ± 8.36 years. For the instruments, the data were appropriate for factor analysis given that Bartlett's tests of sphericity were statistically significant (*p* < .001) and KMO values were quite high (.97).

Factors were extracted using repetitive PCA with orthogonal rotation, and then the same criteria were used for factor retention. The factor loadings of each item in addition to the eigenvalues of each factor, variance explained, cumulative variance, and Cronbach's α of the good nurse questionnaire are shown in Table 1. The same information about the better nursing questionnaire is presented in Table 2. For the good nurse questionnaire, 34 of the 45 items that loaded on the five factors were extracted, explaining 65.6% of the variance, whereas 26 of the 45 items that loaded on the three factors were identified for better nursing, explaining 67.1% of the variance. No items were cross-loaded or had a factor loading value less than .50.

**Table 1:** Primitive Construct of the Good Nurse Questionnaire Using Exploratory Factor Analysis and Reliability Analysis

Item		Mean	*SD*	Factor Loading				
				1	2	3	4	5
**A good nurse…**								
**1.**	**helps fellow nurses keep up with the latest expertise.^a^**	4.23	.71	.75	.23	.22	.16	.04
**2.**	focuses on what patients need now by paying careful attention.	4.19	.68	.74	.26	.21	.18	.09
**3.**	respects the expectations and culture of the patient.	4.16	.71	.73	.23	.26	.19	−.03
**4.**	**maintains a good relationship with the staffs of other departments based on an understanding of their work.^a^**	4.21	.65	.72	.31	.19	.21	.09
**5.**	**empathizes with the difficulties of assistance personnel in the hospital and has a warm and respectful attitude.^a^**	4.25	.68	.69	.22	.24	.13	.24
**6.**	**finds the strengths of fellow nurses and praises them rather than criticizes.^a^**	4.17	.70	.69	.21	.16	.16	.14
**7.**	provides all necessary information to patients/guardians as fully as possible even when s/he is busy.	4.24	.68	.69	.26	.22	.10	.17
**8.**	establishes trust with various medical staffs.	4.24	.67	.66	.27	.21	.17	.14
**9.**	reflects on whether nursing care she/he has provided is good enough.	4.17	.68	.66	.33	.13	.18	.24
**10.**	acknowledges that there was a lack of nursing provided to the patient when the patient/guardian complains.	4.07	.71	.65	.10	.10	.15	.39
**11.**	actively approaches nervous patients and helps them calm down.	4.14	.71	.64	.32	.03	.16	.30
**12.**	**works with her or his nurse colleagues, helping one another.^a^**	4.41	.63	.63	.28	.32	.18	−.03
**13.**	coordinates health examinations and appointments to promote patient comfort.	4.03	.74	.56	.26	.02	.13	.36
**14.**	**works fast enough to ensure smooth workflow.^a^**	**4.27**	**.66**	**.31**	**.74**	**.21**	**.26**	**.12**
**15.**	responds quickly to the reports of the patient/family.	4.29	.66	.32	.74	.20	.18	.17
**16.**	**thoroughly prepares in advance and constantly checks on the safety of medical treatments.^a^**	4.37	.64	.35	.73	.27	.05	.12
**17.**	assesses the patient's critical symptoms in time and informs the doctor, if necessary.	4.47	.61	.28	.71	.30	.09	.09
**18.**	**performs proper nursing practices according to my role and work.^a^**	4.30	.62	.26	.58	.24	.28	.18
**19.**	is trusted by colleagues because (s)he is devoted to her or his work.	4.24	.66	.35	.58	.30	.33	.05
**20.**	**communicates well with other departments and professionals.^a^**	4.29	.65	.40	.58	.28	.20	.14
**21.**	gains self-confidence through her or his personal growth experience.	4.13	.73	.37	.58	.14	.37	.17
**22.**	**talks to patients in a way they can understand.^a^**	4.33	.65	.32	.55	.37	.14	.14
**23.**	cares for patients based on professional ethics.	4.46	.65	.23	.36	.69	.13	.14
**24.**	cares for patients conscientiously and honestly.	4.51	.62	.18	.39	.67	.11	.16
**25.**	listens to patients/guardians carefully and answers their questions sincerely.	4.36	.62	.23	.29	.64	.20	.15
**26.**	**does job with a bright and energetic attitude.^a^**	4.23	.67	.26	.20	.62	.24	.17
**27.**	**believes that s/he can make the most of her or his ability.^a^**	4.14	.70	.32	.21	.61	.29	.08
**28.**	**is satisfied and happy with working as a nurse.^a^**	3.78	.90	.20	.18	.14	.81	.17
**29.**	**feels proud and has a sense of achievement after completing a lot of daily work.^a^**	4.02	.81	.22	.21	.18	.78	.17
**30.**	**feels happy for being helpful to others, even if nobody acknowledges.^a^**	3.94	.85	.26	.22	.15	.74	.24
**31.**	**thinks the nursing profession is valuable.^a^**	**4.28**	**.82**	**.21**	**.21**	**.36**	**.63**	**.10**
**32.**	**has compassion for patients/their families.^a^**	3.92	.88	.22	.16	.03	.20	.75
**33.**	**feels bad when unable to take care of a patient's request.^a^**	4.13	.70	.20	.19	.39	.14	.62
**34.**	**shares experiences that can help the patient/guardian.^a^**	3.94	.79	.19	.12	.31	.24	.62
Eigenvalue				7.6	5.3	3.7	3.4	2.3
Variance explained (%)				22.4	15.7	10.8	9.9	6.8
Cumulative variance (%)				22.4	38.0	48.9	58.8	65.6
Cronbach's α				.94	.93	.86	.87	.73

**^a^**Items were retained after confirmatory factor analysis.

**Table 2:** Primitive Construct of the Better Nursing Questionnaire Using Exploratory Factor Analysis and Reliability Analysis

Item		Mean	*SD*	Factor Loading		
				1	2	3
Better nursing is to…						
**1.**	create a comfortable atmosphere so that the patient can easily talk about his or her concerns.	4.19	.68	.80	.27	.18
**2.**	allow time for the patient to talk.	4.17	.71	.78	.31	.20
**3.**	understand the patient better by listening attentively to what the patient has to say.	4.12	.71	.77	.31	.09
**4.**	**know and practice the importance of holding a patient's hand.^a^**	4.16	.73	.74	.36	.16
**5.**	**spend time reflecting on and fully understanding herself or himself and thereby better reach out to patients.^a^**	4.13	.73	.67	.34	.26
**6.**	**try to fulfill the wishes (advance directives) of terminal patients.^a^**	4.14	.72	.66	.29	.34
**7.**	facilitate family presence with the dying patient.	4.22	.70	.65	.39	.26
**8.**	**take care of patients as her or his own family.^a^**	4.14	.78	.64	.38	.33
**9.**	link resources to help patients with economic difficulties.	4.07	.79	.62	.35	.28
**10.**	**actively comfort the bereaved family.^a^**	4.12	.75	.61	.27	.39
**11.**	**respect and consider as fellow human beings patients who are alienated or vulnerable (e.g., homelessness or alcoholism).^a^**	4.09	.74	.59	.31	.37
**12.**	feel humble self-awareness about life and death as a human being when witnessing the death of a patient.	4.14	.76	.59	.30	.35
**13.**	**treat patients not as objects of work from a disease-oriented perspective but as fellow human beings.^a^**	4.10	.74	.56	.36	.29
**14.**	**feel glad or healed from taking care of patients.^a^**	4.06	.81	.54	.29	.40
**15.**	give attention to the subjective appeals of patients who are in discomfort without relying solely on objective information.	4.27	.71	.32	.74	.15
**16.**	establish, plan, and carry out nursing goals.	4.14	.75	.37	.73	.33
**17.**	**approach patients/guardians with a better understanding through diverse experiences.^a^**	4.30	.66	.29	.72	.28
**18.**	**become an agent for change in terms of fostering a positive relationship with the doctor.^a^**	4.10	.75	.39	.72	.18
**19.**	**understand each patient's unique situation and find the most appropriate method to communicate and approach.^a^**	4.19	.69	.32	.71	.38
**20.**	**actively and creatively seek the most appropriate nursing method for the patient.^a^**	4.12	.76	.36	.70	.36
**21.**	**more actively approach patients who are having difficulties.^a^**	4.23	.69	.39	.68	.28
**22.**	approach the patient with an integrated judgment of the patient's situation.	4.21	.71	.31	.68	.40
**23.**	use a nurse as a therapeutic tool.	4.03	.76	.33	.63	.14
**24.**	**make professional judgments and try to actively resolve situations.^a^**	4.32	.66	.30	.35	.79
**25.**	**accurately and quickly respond to emergencies and unexpected situations.^a^**	4.35	.68	.32	.28	.77
**26.**	**have a certain level of various competences such as responsibility, personality, ethics, and knowledge.^a^**	4.26	.69	.30	.35	.74
Eigenvalue				15.0	1.4	1.0
Variance explained (%)				28.8	24.0	14.3
Cumulative variance (%)				28.8	52.8	67.1
Cronbach's α				.95	.94	.89

^a^Items were retained after confirmatory factor analysis.

### Model Refining and Validating Stage

The hypothesized models of the good nurse questionnaire and better nursing questionnaire from the EFA results were tested using CFA on the second half of the main survey participants (*n* = 468 RNs). Most of the participants in this group were female (95.9%, *n* = 449), were an average of 34.49 ± 9.35 years old, self-identified as religious (57.3%, *n* = 268), worked as a staff nurse (83.3%, *n* = 390), and had an average career duration of 11.01 ± 8.46 years.

#### Good nurse questionnaire

Observational variables of latent construct were examined for reliability and significance using CFA. The model fit indices of the primitive good nurse questionnaire were not satisfactory, as shown in Table 3. Thus, the constructs of the primitive questionnaires from the EFA result were modified using the modification index of CFA. In the modified good nurse model, the number of items was decreased from 34 to 17, and the GFI was improved (normed χ^2^ = 2.14, GFI = .95, AGFI = .92, CFI = .97, NFI = .95, TLI = .96, SRMR = .03, and RMSEA = .05). The χ^2^ test was significant (*p* < .001), indicating that the sample correlation matrix did not fit the hypothesized model. However, the χ^2^ test is particularly sensitive to sample size and is often significant when a large sample is used (Hair et al., 2010). Therefore, examining various fit indices is recommended.

**Table 3:** Model Fit Indices of the Good Nurse Questionnaire and the Better Nursing Questionnaire

Fit Index		Absolute Fit Index							Incremental Fit Index		
		χ^2^	*p*	Normed χ^2^	RMSEA	SRMR	GFI	AGFI	NFI	CFI	TLI
Evaluation criteria		> .05		< 3	≤ .05	≤ .08	≥ .9	≥ .9	≥ .9	≥ .9	≥ .9
A good nurse	Original model (34 items)	1701.76	< .001	3.29	.07	.05	.80	.76	.85	.89	.88
		*df* = 517									
	Modified model (17 items)	232.97	< .001	2.14	.05	.03	.95	.92	.95	.97	.96
		*df* = 109									
Better nursing	Original model (26 items)	1090.62	< .001	3.69	.05	.03	.84	.81	.90	.93	.92
		*df* = 296									
	Modified model (16 items)	207.56	< .001	2.06	.05	.03	.95	.93	.97	.98	.98
		*df* = 101									

***Note*.** RMSEA = root mean square error of approximation; SRMR = standardized root mean squared residual; GFI = goodness of fit index; AGFI = adjusted goodness of fit index; NFI = normed fit index; CFI = comparative fit index; TLI = Tucker–Lewis index.

As shown in Table 4, the factor loadings of the 17 items ranged between .57 and .87, indicating statistical significance (*p* < .001). The convergent validity of the final model was acceptable considering that the AVE values ranged from .46 to .63, higher than a criterion of .5, with the exception of one factor that exhibited moderate convergent validity. Moreover, conceptual reliability ranged from .71 to .89, which was higher than the minimum acceptable level of .7, indicating convergent reliability.

**Table 4:** Final Construct of the Good Nurse Questionnaire Using Confirmatory Factor Analysis and Reliability Analysis

Questionnaire/Factor	Item	Mean	*SD*	λ	SMC	CR	AVE	Cronbach's α
A good nurse								
Factor 1						.89	.62	.89
	4	4.18	0.67	.87	.75			
	6	4.16	0.69	.81	.66			
	5	4.21	0.69	.78	.61			
	12	4.42	0.63	.73	.54			
	1	4.18	0.72	.73	.54			
Factor 2						.85	.59	.79
	16	4.30	0.66	.73	.53			
	18	4.24	0.64	.77	.59			
	22	4.32	0.66	.76	.58			
	20	4.18	0.75	.82	.68			
Factor 3						.71	.55	.71
	27	4.15	0.69	.74	.55			
	26	4.23	0.71	.75	.56			
Factor 4						.83	.63	.83
	29	4.01	0.79	.80	.64			
	30	3.91	0.86	.84	.70			
	28	3.75	0.92	.73	.54			
Factor 5						.71	.46	70
	33	4.12	0.69	.75	.56			
	34	3.94	0.83	.70	.49			
	32	3.93	0.82	.57	.32			
Total								.93
Better nursing								
Factor 1						.93	.61	.93
	14	4.03	0.83	.75	.56			
	13	4.11	0.76	.77	.60			
	11	4.13	0.76	.81	.66			
	6	4.11	0.76	.82	.67			
	10	4.07	0.79	.79	.62			
	8	4.15	0.74	.80	.63			
	4	4.18	0.75	.78	.61			
	5	4.08	0.76	.75	.56			
Factor 2						.91	.66	.91
	20	4.13	0.75	.85	.71			
	19	4.21	0.69	.86	.74			
	18	4.03	0.77	.77	.59			
	21	4.20	0.69	.80	.64			
	17	4.22	0.71	.80	.64			
Factor 3						.90	.74	.90
	25	4.33	0.70	.83	.69			
	24	4.30	0.67	.89	.78			
	26	4.23	0.71	.87	.76			
Total								.96

***Note*.** All factor loadings were statistically significant at the *p* < .001 level. λ = standardized factor loading; SMC = square multiple correlation; CR = conceptual reliability; AVE = average variance extraction.

To assess discriminant validity, the confidence interval of the estimated correlation between factors was calculated. Whereas factor correlations ranged between .66 (Factor 1 and Factor 5) and .84 (Factor 3 and Factor 4), the confidence interval of the estimated correlations between Factor 3 and Factor 4 ranged from .78 and .90 and did not include 1 (Haddock & Maio, 2004). The five constructs of the final model were considered validly discriminant.

After reviewing the CFA results of a good nurse questionnaire with regard to its use as a tool to measure the virtues of good nurses, the final measurement model consisted of five factors: collaboration (five items), professional competency (four items), self-efficacy (two items), a sense of achievement (three items), and compassion (three items). The Cronbach's alpha for internal consistency was .93, ranging from .70 to .89, depending on a factor of a good nurse. The definition for each factor is provided in Table 5.

#### Better nursing questionnaire

The model fit indices of the primitive questionnaire of the EFA results for better nursing were not satisfactory, as shown in Table 3, and the model was modified using the CFA. In the modified questionnaire model, the number of items was reduced from 26 to 16, and the GFIs were improved as follows: normed χ^2^ = 2.06, GFI = .95, AGFI = .93, CFI = .98, NFI = .97, TLI = .98, SRMR = .03, and RMSEA = .05.

As shown in Table 4, the factor loadings of the 16 items ranged between .75 and .89 (*p* < .001). The AVE values were .61–.74 (> .5), and convergent validity was acceptable. Conceptual reliability ranged between .90 and .93 (> .7), indicating good convergent reliability. Factor correlations ranged between .86 (Factor 2 and Factor 3) and .89 (Factor 1 and Factor 2). The confidence interval for the estimated correlation between Factor 1 and Factor 2 (.83 and .95) did not include 1. Thus, the discriminant validity of the final model was considered acceptable.

After reviewing the CFA results of the better nursing questionnaire, the final measurement model included three factors: person-centered nursing (eight items), proactive nursing (five items), and expertise in nursing (three items). The Cronbach's alpha for internal consistency was .96, ranging from .90 to .93. The definitions of each factor are provided in Table 5.

**Table 5:** Descriptions of the Questionnaires' Dimensions

Questionnaire	Dimension	Description
A good nurse		
Factor 1	Collaboration	Establishing a collaborative relationship with nurses and other personnel
Factor 2	Professional competency	Playing a diverse nurse role based on professional knowledge and skills
Factor 3	Self-efficacy	Believing one's own ability with a positive attitude
Factor 4	A sense of achievement	Having a strong sense of pride and satisfaction in one's nursing care
Factor 5	Compassion	Having empathy to patients and supporting them
Better nursing		
Factor 1	Person-centered nursing	Treating a patient with respect as a whole person, preserving personhood
Factor 2	Proactive nursing	Carrying out proactive roles for caring a patient
Factor 3	Expertise in nursing	Providing care in a fluid and seamless manner

## Discussion

The constructs of a good nurse questionnaire and better nursing questionnaire were validated by conducting exploratory and confirmatory analyses on nationwide survey data of RNs in Korea. In this section, the five constructs of the good nurse questionnaire will be discussed first, followed by a discussion of the features of the better nursing questionnaire. On the basis of the results of this study, a good nurse may be defined as a professional who performs her or his role well in terms of the essential five virtues of “collaboration, professional competency, self-efficacy, a sense of achievement, and compassion.”

The first construct of the good nurse questionnaire, “collaboration,” was also recognized as an attribute of a good nurse from the perspective of nurses in a previous study (Catlett & Lovan, 2011; Kim et al., 2019). It may be difficult for patients to be aware of how collaboration among nurses influences their care. The modern code of ethics for nurses emphasizes collaboration with other healthcare professionals based on mutual trust and respect (International Council of Nurses, 2012; Korean Nurses Association, 2013). Collaboration is an ethical responsibility of nurses because quality care and patient safety are possible in today's complex healthcare environments through interdisciplinary care teams working in collaboration with multiple healthcare providers and through nurses creating a bridge between these care teams and patients. Nurses relay patient information to other nurses, doctors, physiotherapists, social workers, dieticians, and other healthcare professionals. Thus, the nursing virtue of collaboration has become even more critical.

The two virtues of “professional competency” and “compassion” on the good nurse questionnaire were frequently identified in previous studies both by nurses (Aydin Er et al., 2017; Catlett & Lovan, 2011; Kim et al., 2019) and by patients (Rchaidia et al., 2009; Van der Elst et al., 2012). There is no question that a good nurse should present appropriate professional knowledge, skills, and attitudes with compassion. Nurses must continuously improve their knowledge and skills as healthcare professionals to adopt new treatments and better technology for the benefit of their patients. At the same time, a good nurse must be able to feel empathy toward patients as well as support and build therapeutic relationships with their patients (Gastmans et al., 1998). A nurse who has excellent nursing knowledge and skills but lacks a compassionate attitude may not be considered to be a good nurse.

Finally, “self-efficacy” and “a sense of achievement” were identified as critical virtues of a good nurse. A good nurse not only believes in his or her own ability with a positive attitude for patient care but also possesses a strong sense of achievement based on professional pride and satisfaction in his or her nursing practice. These psychological attributes are common characteristics of professionals (Evetts, 2014). Nursing care is a professional response that is customized to a wide array of difficult patient conditions, which may be interpreted quite differently depending on an individual nurse's self-efficacy. In the current healthcare environment, nurses are required to confront and manage diverse challenges. Thus, nurses must possess a high self-efficacy. Furthermore, patients in a Japanese study (Izumi et al., 2006) expressed that they expected a good nurse to have pride in and a passion for nursing work. This study also found that good nurses felt pride and happiness in their nursing work, which related closely to their professionalism. In particular, nurses' professionalism in terms of valuing their work may lead them to remain in their profession and workplace (Çelik & Hisar, 2012; Guerrero et al., 2017). Because of concerns over high rates of professional attrition, sense of achievement has been increasingly considered to be a key virtue in the nursing profession. In conclusion, a good nurse must be able to provide professional care with a compassionate attitude, collaborate with diverse work teams, and present a high level of self-efficacy and sense of achievement.

Three aspects characterize the concept of better nursing, including, in descending order of explained variance, “person-centered nursing” (28.8%), “proactive nursing” (24.0%), and “expertise in nursing” (14.3%). Of nursing practices exhibited by good nurses, better nursing in this study referred to a nurse who performs at a higher level than expected. As anticipated, one of the better-nursing characteristics, “expertise in nursing,” facilitates the integration by nurses of professional knowledge and skills into actual practice with a deep understanding of a clinical situation based on their experience. However, this factor explained only 14.3% of the concept.

“Person-centered nursing” and “proactive nursing” largely explained the concept of better nursing. In line with “person-centered nursing,” nurses are expected to be nonjudgmental and respectful of each patient's life, preserve the personhood of their patients, and feel that they are healed through the therapeutic relationship. Currently in nursing, person-centered care is defined as a holistic approach that provides respectful and individualized care to patients (Morgan & Yoder, 2012). The more that medical treatment depends on high technology to maximize efficiency and accuracy, the more that patients desire to seek humanity in nursing care. Nurses should pursue person-centered care continuously in their own nursing practices without compromising on nursing care quality. Although person-centered care may not be challenging for most patients, it becomes critical for those patients with special needs or complex conditions. Exceptional efforts are required for a nurse to be responsive to and responsible for those patients. Therefore, the concept of person-centered care may not be considered beyond “proactive nursing” in better nursing. “Proactive nursing” means taking an active approach toward patients to address individualized care needs using creative problem-solving abilities and playing a role as an agent for change in the treatment team.

Better nursing practices were highlighted in exemplars cited by Benner (1984). She asserted that these exemplars described excellence and power in nursing, including transformative power, integrative caring, advocacy, healing power, affirmative power, and creative problem solving. Better nursing may require the virtues and excellence of a good nurse. According to Aristotle's virtue ethics, virtue grows mostly from teaching and results from habit. Therefore, being a good nurse is a process that requires teaching, repeated practice, and then habituation in one's work. This study has practical and educational implications. The five essential virtues of a good nurse may be used to guide a prelicensure nursing education program. To train up good nurses, nursing education should cultivate all five of these virtues, which should be taught via classroom and extracurricular activities.

The virtues of a good nurse and the characteristics of better nursing may be used to evaluate nurses and their practice in nursing organizations. Most nursing instruments assess either nursing competencies or caring behaviors separately in relation to a good nurse or better nursing (Park & Kim, 2016). However, being a good nurse and acting better nursing may require both competent practice and caring behavior. Furthermore, nursing professional development must focus on “what sort of nurse they ought to be,” while admitting the importance of learning “what and how to do” for the nursing profession. The concepts of the good nurse questionnaire and better nursing questionnaire may help expose nurses to a good role model for clinical practice in terms of “what sort of nurse they ought to be” as well as “what and how to do.” Therefore, the questionnaires may be useful for the evaluation of nurses and their practice in a holistic way and for guiding nurses to reinforce their strengths and improve their weaknesses in terms of being a good nurse and acting better nursing.

The study findings are meaningful given that Korean nurses' understandings of a good nurse and better nursing have never been formally discussed and agreed upon until now. It is timely to have formulated these agreed-upon concepts because of the increasing diversity in the nursing workforce in terms of age, gender, academic background, and previous work experiences. However, the results of this study should be considered in the cultural context of Korean nursing. Because the role and status of nurses vary from country to country, the concept of Korean nurses of “good nurse” and “better nursing” may not be generalizable to other countries. However, on the basis of the findings of previous studies, the characteristics of these two concepts seem to share much in common across countries. If the questionnaires are used in different countries, nursing scholars may compare the related concepts and better understand how different nursing systems and roles influence the concept of a good nurse and better nursing. In addition, these two concepts are changing over time alongside changes in the role of nurses. Therefore, continued study of these concepts is necessary.
